# Exploring Social Contextual Correlates of Computer Ownership and Frequency of Use Among Urban, Low-Income, Public Housing Adult Residents

**DOI:** 10.2196/jmir.9.4.e35

**Published:** 2007-12-13

**Authors:** Lorna H McNeill, Elaine Puleo, Gary G Bennett, Karen M Emmons

**Affiliations:** ^3^Center for Community-based ResearchDana-Farber Cancer Instituteand Department of SocietyHuman Development and HealthHarvard School of Public HealthBostonMAUSA; ^2^Department of BiostatisticsSchool of Public Health and Health SciencesUniversity of Massachusetts AmherstAmherstMAUSA; ^1^Department of Health Disparities ResearchThe University of Texas MD Anderson Cancer CenterHoustonTXUSA

**Keywords:** Computers, minority groups, African American, Hispanics, social context, social environment, low-income population, socioeconomic position, social networks, neighborhoods

## Abstract

**Background:**

As advances in computer access continue to be made, there is a need to better understand the challenges of increasing access for racial/ethnic minorities, particularly among those with lower incomes. Larger social contextual factors, such as social networks and neighborhood factors, may influence computer ownership and the number of places where individuals have access to computers.

**Objectives:**

We examined the associations of sociodemographic and social contextual factors with computer ownership and frequency of use among 1554 adults living in urban public housing.

**Methods:**

Bivariate associations between dependent variables (computer ownership and regular computer use) and independent variables were used to build multivariable logistic models adjusted for age and site clusters.

**Results:**

Participants (N = total weighted size of 2270) were on average 51.0 (± 21.4) years old, primarily African American or Hispanic, and earned less than US $20000 per year. More than half owned a computer, and 42% were regular computer users. Reporting computer ownership was more likely if participants lived above the poverty level (OR = 1.78, 95% CI = 1.39-2.29), completed high school (OR = 2.46, 95% CI = 1.70-3.55), were in financial hardship (OR = 1.38, 95% CI = 1.06-1.81), were employed and supervised others (OR = 1.94, 95% CI = 1.08-3.46), and had multiple role responsibilities (OR = 2.18, 95% CI = 1.31-3.61). Regular computer use was more likely if participants were non-Hispanic (OR = 1.94, 95% CI = 1.30-2.91), lived above the poverty level (OR = 2.84, 95% CI = 1.90-4.24), completed high school (OR = 4.43, 95% CI = 3.04-6.46), were employed and supervised others (OR = 2.41, 95% CI = 1.37-4.22), felt safe in their neighborhood (OR = 1.57, 95% CI = 1.08-2.30), and had greater social network ties (OR = 3.09, 95% CI = 1.26-7.59).

**Conclusions:**

Disparities in computer ownership and use are narrowing, even among those with very low incomes; however, identifying factors that contribute to disparities in access for these groups will be necessary to ensure the efficacy of future technology-based interventions. A unique finding of our study is that it may be equally as important to consider specific social contextual factors when trying to increase access and use among low-income minorities, such as social network ties, household responsibilities, and neighborhood safety.

## Introduction

There has been a growing emphasis on technology-based strategies to increase reach, efficacy, sustainability, and cost-effectiveness of preventive health interventions. Communication strategies, many of which utilize computers and the Internet, are being recognized as potential modalities for reducing health disparities via the dissemination of culturally appropriate health information to racial/ethnic minorities and low-income populations [1].

Certainly, disparities in health outcomes can be attributed to cultural and societal factors, such as access to health care [2], but health disparities in the United States are likely influenced by a lack of access to health information [3]. A 2000 report by the Pew Internet & American Life Project noted that racial/ethnic minorities and those in lower income groups are interested in using computers and the Internet to access health information [4]. More recent findings from the Health Information National Trends Survey (HINTS) show that more than 60% of black and 56% of Hispanic online users looked for health or medical information online [5]. National data suggest that computer ownership among racial/ethnic minorities is increasing, although it is still less compared to whites. In 2003, 64% of whites reported having one or more computers in the home; the number of African American and Hispanic households with a computer was 45% and 44%, respectively [6]. Likewise, among those with very low incomes (< US $20000 per year), studies have shown that ownership is greater for whites compared to African Americans and Hispanics [7].

As advances in computer access continue to be made, there is a need to better understand the challenges of increasing access for racial/ethnic minorities, particularly among those with lower incomes. It is well known that access to communication technologies is differentially associated with social class. For example, income, education, and employment are positively associated with subscriptions to Internet services and newspapers [3]. However, the influence of social contextual factors on computer use, specifically computer access, still needs to be determined.

Social contextual factors are those that shape an individual’s day-to-day experience, such as one’s neighborhood or work environment as well as social norms of health and behavior [8-10]. It is likely in this regard that in addition to socioeconomic resources a combination of other factors, such as personal time constraints and multiple role responsibilities (eg, caregiving responsibilities) [11], as well as larger societal forces, such as social network ties and neighborhood factors, influence computer ownership and the number of places where individuals have access to computers. This may be particularly true for lower income groups who live in poor neighborhoods. For example, a 2003 report by the Public Access Computing Project [12] found that while low-income families living in lower income neighborhoods and low-income families living in higher income neighborhoods reported computer use at similar rates (~ 58%), low-income families living in lower income neighborhoods reported slightly less computer ownership than their counterparts living in higher income neighborhoods. Access to computers can potentially link disenfranchised communities to greater informational, social, and economic resources, thereby potentially building neighborhood social capital [13].

There have been very few studies of the association between social contextual factors and computer access and use. This is an important omission because we posit that attempts to reduce communication disparities may fail if focused solely on sociodemographic factors. Therefore, this study examines the combination of sociodemographic and social contextual factors and their influence on computer ownership and frequency of use among adults living in urban public housing.

## Methods

This study uses baseline data from an ongoing randomized controlled trial of a colorectal cancer prevention intervention, “Open Doors to Health,” conducted in 12 urban subsidized housing complexes in Boston, MA, United States.

The housing site is the unit of randomization and intervention. Unequal probability sampling was used because of the varying size of housing sites. In the sites that had a population of less than 300, all adult residents were sampled. In the remaining sites, with a population greater than 300 adult residents, researchers obtained a 35% sample, with a minimum of 250 participants per site. Sites were matched for randomization to intervention condition based on population size, ethnicity ratio, and age group ratio (≤ 50 years, > 50 years) when possible.

### Conceptual Model

**Figure 1:**
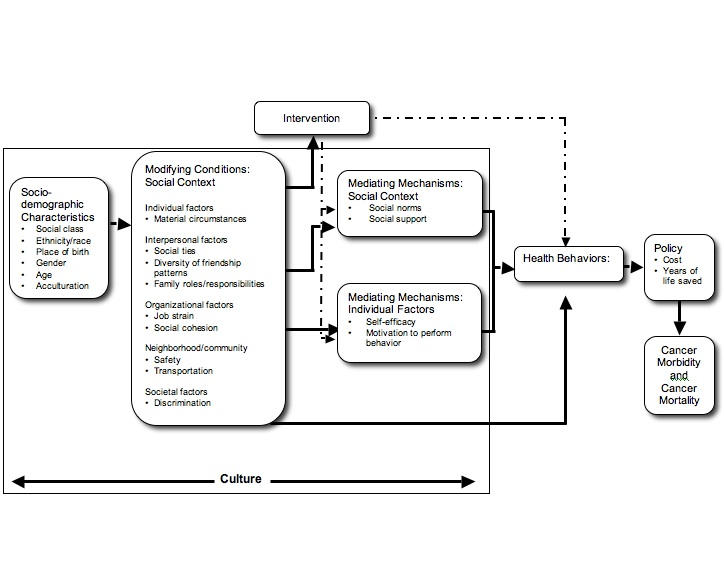
Conceptual model

Figure 1 depicts a conceptual framework that explicates the role of the social context in health behavior change [9]. We chose a social ecological framework to illustrate social contextual factors across multiple levels of influence [14-17]. Among these were individual factors, which include material circumstances such as owning one’s own car or having adequate resources for child care. Interpersonal factors, such as the presence of social ties, family roles and responsibilities, and social norms, are likely to be powerful correlates of health behaviors and may vary by factors reflecting cultural differences (race/ethnicity, acculturation). Organizational factors may reflect the work setting, for example, job stress, control, and exposure to a hazardous work environment. Neighborhood and community factors measured on an individual level include access to a safe neighborhood. Finally, larger societal forces, such as racial discrimination, may also shape health behaviors and outcomes.

Social contextual factors, in turn, may influence health behaviors directly or indirectly through individual psychosocial factors. Social cognitive theory [18,19], the theory of reasoned action [20,21], and the transtheoretical model of behavior change [22,23] are guiding models that highlight specific individual psychosocial factors that predict a change in behavior. Psychosocial mediating variables in large part influence intentions to change behavior, which are highly associated with the likelihood of change [24,25].

One’s social context and day-to-day realities are shaped by sociodemographic characteristics, which may influence a range of interrelated health behaviors. For example, socioeconomic position, race and ethnicity, nativity, gender, and age are important correlates of health outcomes. Identifying disparities in health behaviors across populations with these characteristics can inform priority setting and guide policy decisions. In addition, culture, that is the learned and shared knowledge and beliefs used to interpret experiences, cuts across all domains in this model [26,27].

### Study Recruitment

Recruitment for Open Doors to Health began in 2004. Participants provided informed consent and completed an interviewer-administered survey in either English or Spanish. Participants received US $25 compensation. Eligibility criteria for the study survey included (1) living in the housing community, (2) being at least 18 years old, (3) being fluent in English or Spanish, and (4) not having cancer. An initial sample of 3688 subjects was drawn. Of them, 747 (20%) were deemed ineligible, leaving 2941 eligible individuals. Of these, 828 (28%) refused participation, and 559 (19%) could not be reached, leaving 1554 residents who completed the baseline survey. This yielded an overall 53% response rate, with a range of 34% to 92% across the housing sites. The study protocol was approved by the Human Subjects Committee at the Harvard School of Public Health.

### Sociodemographic Characteristics

Sociodemographic variables collected included gender, date of birth, race/ethnicity (categorized as black, white, Hispanic, and other), and highest level of education completed. We also assessed poverty status and financial situation with two measures. Yearly household income (six response options ranging from less than US $10000 to at least US $50000) and the number of people supported by this income were used to measure poverty status (dichotomized as being above or below the poverty level based on the 2005 federal poverty guidelines on income and household size) [28]. Participants were also asked about their perception of the financial status of their household (comfortable with some extras, enough but no extras, have to cut back, or cannot make ends meet).

We assessed employment status in several ways. Participants were asked if they were working, and, if so, (1) whether they worked full-time or part-time and (2) the number of hours worked in a week, including overtime or extra hours. Hours worked were categorized as 0, less than 20, 20 to < 37, and 37+ hours per week. Participants were also asked about the number of jobs (beyond their main job) they worked (0, 1, or more than 1) and whether they supervised employees.

Lastly, we assessed immigrant status by asking participants their birthplace, the number of years they lived in the United States, and their first or native language.

### Social Contextual Factors

Each participant was asked about several social contextual factors. Neighborhood safety was assessed by asking whether participants felt safe walking alone in their neighborhood during the day and at night [29]. For both daytime and nighttime, participants were asked “How safe do you feel walking alone in your neighborhood?” Response options included “safe,” “a little unsafe,” and “unsafe.” For analysis purposes, we combined the response categories of “a little unsafe” and “unsafe” for daytime safety due to the small number of responses in the latter category.

To assess social cohesion in the housing community, we asked respondents to report their agreement with five statements: (1) people around here are willing to help their neighbors; (2) this is a close-knit neighborhood; (3) people in this neighborhood can be trusted; (4) people in this neighborhood generally do not get along with each other; and (5) people in this neighborhood do not share the same values. Item responses were reversed for the first three statements and then responses to the five items were averaged. The summary score ranged from 1 to 4, with a higher score indicating higher social cohesion [30].

Marital status, number of close friends, number of close family members, and active membership in organizations (religious, professional, community, civic, etc) were combined to form a continuous measure of the number of social network ties ranging from 0 to 4, with a higher score indicating a greater social network [31]. Social support was assessed by asking participants about emotional support from family and friends, support when sick, help with household tasks, financial support, and help getting to the doctor. Responses to questions of social support were measured on a 5-point Likert scale ranging from “yes, always have someone to help” to “no, no one like that.” A single social support variable was created by adding the number of responses to the five questions that indicated at least some support, with a range of 0 to 5. Higher scores indicated greater social support [32].

Participants were asked about their various family roles, which included “earning money to support the family,” “taking care of children,” and “taking care of another household.” The measure of multiple roles was computed as the number of family roles for which the participant was mostly or fully responsible (0 to 3). To determine role conflicts, participants were asked whether their daily activities made conflicting demands on them (ie, role conflict) [9].

Health status was captured by asking participants whether health problems make it difficult for them to exercise (yes/no).

Participants were also asked to report the number of hours per day (during the week and weekend) that they watched television  [33].

### Computer Ownership and Use

We assessed computer ownership and frequency of use (daily, weekly, monthly, less than monthly, and never). Use was recoded as regular (daily and weekly), intermittent (monthly and less than monthly), and never. For multivariable modeling purposes, this variable was further dichotomized as regular versus intermittent or no use. Participants were also asked where they most often use a computer: home, work, housing site, library, friend’s house, community center, or other. The latter five response options were coded as “other” for the purpose of these analyses.

### Data Analysis

On the basis of the cluster design, data for all analyses were weighted up to the population size within each housing site (with a total weighted size of 2270). Frequency distributions and estimates of means and standard deviations were assessed for distributional assumptions and outliers. Bivariate associations between the dependent variables, computer ownership and use of a computer, and independent variables were assessed, and variables found to be significant at the *P* = .15 level in bivariate analyses were retained for use in multivariable modeling. Based on the bivariate associations and consideration of effect modifiers (ie, interaction effects) and confounders, multivariable logistic models of the dependent variables were developed. Bivariate associations with computer use were assessed using three category polytomous logistic regression models with a generalized logit assumption. Based on the assessment of these models, the sample size of the intermittent users and consideration of cluster model constraints, we dichotomized the computer use variable to regular versus intermittent and nonusers. Thus, all multivariable models are dichotomous logistic models. All analyses were conducted to adjust for age as a potential confounder. All analyses were conducted using SUDAAN version 9.0.1 (Research Triangle Institute, Research Triangle Park, NC, USA) and SAS statistical software version 9.1 (SAS Institute, Cary, NC, USA) for clustered data.

## Results

Sociodemographic characteristics and social contextual factors by computer ownership, frequency of use, and location of use are shown in Table 1.

### Sociodemographic Characteristics

The majority of the study participants were female (74%), not working or disabled (63%), and earned less than US $20000 per year (74%). The mean age of the participants was 51.0 ± 21.4 years. Almost half of the participants were black (43%), and an equal number were Hispanic (43%); 52% of participants were born in the United States. A slight majority of participants lived above the poverty level (51%); however, 43% considered themselves to be under financial hardship.

### Computer Ownership and Use

More than half (51%) of participants owned a computer, and 42% reported regular computer use (Table 1); 50% of regular users used the computer most often at home. Computer ownership was highest (greater than 70%) among participants who were less than 49 years old, those with at least some college education, and those who were employed. A large number of adults 65+ (86%) and those below the poverty level (58%) had never used a computer. Unemployed participants were more likely to use a computer in places such as a library or friend’s house than at home.

**Table 1:** Computer use weighted frequencies

		**Own a Computer**		**Use a Computer**			**Location of Computer Use**		
		**Yes, No. (%)**	**No, No. (%)**	**Never, No. (%)**	**Intermittent, No. (%)**	**Regularly, No. (%)**	**Home, No. (%)**	**Work, No. (%)**	**Other, No. (%)**
**Overall**		1079 (51.23)	1027 (48.76)	1021 (48.14)	217 (10.23)	882 (41.58)	550 (50.00)	270 (24.55)	280 (25.45)
**Sociodemographics**									
Gender									
	Male	227 (42.47)	307 (57.53)	291 (53.76)	35 (6.55)	215 (39.67)	146 (57.30)	37 (14.52)	72 (28.18)
	Female	852 (54.21)	720 (45.79)	730 (46.24)	181 (11.49)	667 (42.27)	404 (47.85)	233 (27.54)	208 (24.60)
Age (years)									
	< 35	341 (70.73)	141 (29.27)	58 (12.14)	58 (12.04)	365 (75.82)	213 (50.42)	88 (20.81)	122 (28.77)
	35-49	383 (72.44)	146 (27.55)	173 (32.69)	82 (15.55)	273 (51.77)	182 (50.82)	110 (30.87)	65 (18.31)
	50-64	279 (41.31)	396 (58.69)	416 (61.39)	61 (9.03)	201 (29.58)	123 (47.27)	68 (26.03)	70 (26.70)
	65+	77 (18.28)	345 (81.72)	374 (86.42)	16 (3.60)	43 (9.98)	32 (54.86)	3 (5.95)	23 (39.19)
Poverty level									
	Below poverty level	431 (42.61)	580 (57.39)	591 (58.14)	138 (13.59)	288 (28.27)	230 (53.69)	43 (10.06)	155 (36.24)
	Above poverty level	527 (59.35)	361 (40.65)	342 (38.18)	64 (7.11)	490 (54.71)	254 (46.07)	205 (37.04)	93 (16.89)
Financial status									
	Comfortable/enough	589 (50.63)	574 (49.37)	549 (46.72)	105 (8.92)	521 (44.36)	299 (47.94)	177 (28.41)	148 (23.66)
	Have to cut back/can’t make ends meet	466 (52.79)	417 (47.21)	428 (48.57)	108 (12.30)	345 (39.13)	238 (52.10)	93 (20.26)	126 (27.64)
Education									
	≤ 8th grade	92 (20.05)	366 (79.95)	423 (90.94)	18 (3.79)	24 (5.26)	20 (47.16)	4 (10.03)	18 (42.81)
	Some high school	164 (42.64)	221 (57.36)	248 (64.05)	39 (10.21)	100 (25.75)	83 (58.85)	18 (12.50)	40 (28.66)
	Completed high school/vocational	338 (58.93)	235 (41.07)	237 (41.02)	77 (13.29)	264 (46.67)	165 (48.19)	90 (26.31)	87 (25.50)
	At least some college	483 (70.59)	201 (29.41)	111 (16.22)	82 (11.97)	492 (71.81)	281 (49.23)	158 (27.70)	132 (23.07)
Immigrant									
	No	623 (55.40)	501 (44.60)	407 (35.91)	140 (12.35)	586 (51.74)	348 (48.09)	178 (24.64)	197 (27.27)
	Yes	455 (46.47)	524 (53.53)	613 (62.23)	77 (7.82)	295 (29.94)	201 (53.61)	92 (24.40)	82 (21.98)
English 1st language									
	No	471 (48.13)	507 (51.86)	588 (59.81)	78 (7.98)	317 (32.21)	216 (54.37)	100 (25.13)	81 (20.50)
	Yes	607 (53.93)	519 (46.07)	432 (38.06)	138 (12.21)	564 (49.74)	333 (47.48)	170 (24.24)	198 (28.28)
Race/ethnicity									
	Hispanic	425 (46.03)	499 (53.97)	570 (61.19)	74 (7.97)	287 (30.84)	192 (52.63)	90 (24.58)	83 (22.79)
	Black	496 (54.41)	415 (45.59)	343 (37.54)	123 (13.42)	448 (49.04)	263 (46.43)	136 (24.07)	167 (29.50)
	White	41 (50.91)	39 (49.09)	38 (44.93)	4 (4.59)	42 (50.48)	30 (61.98)	12 (23.92)	7 (14.10)
	Other	111 (61.78)	69 (38.22)	68 (37.65)	16 (8.97)	96 (53.39)	59 (52.82)	31 (27.84)	22 (19.33)
**Employment Status**									
Work status									
	Employed	556 (71.46)	222 (28.54)	201 (25.84)	76 (9.79)	501 (64.37)	243 (42.34)	268 (46.59)	64 (11.08)
	Unemployed	158 (57.08)	119 (42.92)	72 (26.22)	51 (18.45)	152 (55.33)	104 (50.63)	1 (0.50)	100 (48.86)
	Not working	366 (34.82)	684 (65.18)	746 (70.01)	90 (8.45)	231 (21.54)	203 (63.50)	1 (0.32)	116 (36.18)
Hours worked (hours/week)									
	0	521 (39.46)	800 (60.54)	817 (61.18)	139 (10.40)	380 (28.43)	306 (58.72)	1 (0.20)	214 (41.09)
	< 20	69 (67.91)	33 (32.09)	38 (37.63)	14 (13.66)	49 (48.70)	42 (66.89)	8 (12.85)	13 (20.26)
	20 to < 37	198 (71.50)	79 (28.50)	73 (26.28)	25 (9.02)	180 (65.70)	94 (46.52)	77 (37.86)	32 (15.62)
	37+	290 (71.54)	116 (28.45)	93 (22.94)	39 (9.65)	274 (67.41)	51 (34.43)	69 (58.83)	21 (6.74)
Supervisor									
	Unemployed/not working	523 (39.46)	803 (60.54)	818 (61.03)	141 (10.50)	382 (28.47)	307 (58.47)	2 (0.39)	216 (41.14)
	Employed and did not supervise employees	435 (69.25)	193 (30.74)	186 (29.65)	63 (10.10)	379 (60.25)	191 (43.37)	198 (45.07)	51 (11.56)
	Employed and supervised employees	119 (81.19)	28 (18.81)	14 (9.19)	13 (8.61)	121 (82.20)	51 (38.52)	69 (51.93)	13 (9.55)
Number of jobs									
	No jobs	523 (39.46)	803 (60.54)	818 (61.03)	141 (10.50)	382 (28.47)	307 (58.47)	2 (0.39)	216 (41.14)
	One job	494 (70.59)	206 (29.41)	192 (27.38)	70 (9.99)	438 (62.63)	216 (42.70)	231 (45.70)	59 (11.59)
	More than one job	62 (79.19)	16 (20.81)	9 (12.05)	6 (8.03)	63 (79.92)	27 (39.64)	37 (53.07)	5 (7.29)
Employment status									
	Full-time	341 (71.49)	136 (28.51)	111 (23.20)	43 (9.10)	323 (67.69)	126 (34.73)	215 (59.02)	23 (6.25)
	Part-time	738 (45.31)	891 (54.69)	910 (55.40)	173 (10.56)	559 (34.04)	424 (57.62)	55 (7.46)	257 (34.93)
									
**Social Contextual Factors**									
Neighborhood safety									
	Unsafe	141 (47.18)	158 (52.82)	178 (59.56)	30 (10.13)	91 (30.31)	66 (54.23)	25 (20.69)	31 (25.08)
	Safe	897 (53.74)	772 (46.26)	740 (44.07)	182 (10.82)	757 (45.12)	458 (48.78)	240 (25.56)	241 (25.66)
Health problems make it difficult to exercise									
	Yes	409 (43.68)	527 (56.32)	561 (59.39)	93 (9.89)	290 (30.72)	198 (51.44)	70 (18.11)	117 (30.45)
	No	670 (57.33)	499 (42.67)	461 (39.19)	124 (10.51)	591 (50.31)	353(49.36)	199 (27.88)	163 (22.77)
Role conflicts (daily activities make conflicting demands)									
	Yes	437 (57.83)	319 (42.17)	315 (41.63)	83 (11.02)	358 (47.35)	225 (51.12)	120 (27.23)	95 (21.64)
	No	618 (48.29)	662 (51.71)	655 (50.75)	128 (9.93)	508 (39.33)	316 (49.55)	147 (23.02)	175 (27.43)
TV use (hours/day)									
	0	15 (38.75)	24 (61.25)	22 (50.58)	5 (12.89)	16 (36.53)	11 (52.23)	2 (9.75)	8 (38.03)
	> 0 to 2	323 (54.61)	269 (45.39)	280 (46.99)	55 (9.17)	261 (43.84)	152 (48.32)	100 (31.83)	62 (19.85)
	> 2 to 4	409 (53.03)	363 (46.97)	360 (46.33)	83 (10.64)	335 (43.03)	198 (47.37)	107 (25.53)	113 (27.10)
	> 4 to 6	191 (51.26)	182 (48.74)	187 (49.97)	25 (6.77)	162 (43.25)	104 (55.25)	46 (24.64)	38 (20.11)
	> 6	138 (42.62)	186 (57.38)	167 (51.61)	49 (15.03)	108 (33.36)	85 (54.33)	15 (9.35)	57 (36.32)

		**Mean (SE)**	**Mean (SE)**	**Mean (SE)**	**Mean (SE)**	**Mean (SE)**	**Mean (SE)**	**Mean (SE)**	**Mean (SE)**
Social ties/networks (0-4)		2.72 (0.03)	2.59 (0.03)	2.62 (0.03)	2.63 (0.06)	2.72 (0.03)	2.70 (0.04)	2.81 (0.05)	2.60 (0.04)
Social support (0-5)		4.47 (0.03)	4.32 (0.04)	4.27 (0.04)	4.36 (0.04)	4.55 (0.03)	4.54 (0.04)	4.51 (0.06)	4.47 (0.07)
Role responsibilities (0-3)		1.59 (0.02)	1.22 (0.02)	1.27 (0.03)	1.51 (0.03)	1.53 (0.03)	1.54 (0.04)	1.76 (0.04)	1.30 (0.05)
Social cohesion (1-4)		2.41 (0.03)	2.57 (0.03)	2.58 (0.03)	2.50 (0.05)	2.40 (0.03)	2.39 (0.04)	2.42 (0.06)	2.48 (0.05)

#### Bivariate and Multivariable Analyses for Computer Ownership

Table 2 displays the odds ratios and associated 95% confidence intervals for both the bivariate models and the multivariable model. Education (completed high school vs not) and ethnicity (Hispanic vs non-Hispanic) were dichotomized.

Being above poverty (OR = 1.78, 95% CI = 1.39, 2.29), in financial hardship (OR = 1.38, 95% CI = 1.06, 1.81), and having completed high school (OR = 2.46, 95% CI = 1.70, 3.55) were positively associated with computer ownership.  Employment and supervisory role (OR=1.94, 95% CI =1.08, 3.46) was also positively associated with computer ownership.  Finally, having greater financial and caretaking responsibilities were positively associated with owning a computer (OR=2.18, 95% CI=1.31, 3.61).

**Table 2:** Predicting ownership of computer, adjusting for age*

		Bivariate Age-Adjusted OR (95% CI) Yes vs No	Multivariable-Adjusted OR (95% CI)^†^, Yes vs No
**Sociodemographics**		
Gender			
	Male	1.00	
	Female	1.47 (0.98-2.22)	
Poverty level			
	Below poverty level	**0.51 (0.43-0.59)**	1.00
	Above poverty level	1.00	**1.78 (1.39-2.29)**
Financial status			
	Comfortable/enough	1.00	1.00
	Have to cut back/can’t make ends meet	**1.24 (1.06-1.45)**	**1.38 (1.06-1.81)**
Education			
	≤ 8th grade	1.00	
	Some high school	**1.91 (1.31-2.78)**	
	Completed high school/vocational	**3.28 (2.36-4.58)**	
	At least some college	**5.11 (3.18-8.19)**	
			
	Did not complete high school		1.00
	Completed high school		**2.46 (1.70-3.55)**
Immigrant			
	No	1.00	1.00
	Yes	**1.56 (1.25-1.96)**	1.33 (0.98-1.81)
English 1st language			
	No	1.00	
	Yes	**3.07 (2.15-4.38)**	
Race/ethnicity			
	Hispanic	1.00	
	Black	1.43 (0.99-2.08)	
	White	**1.62 (1.00-2.62)**	
	Other	**2.21 (1.29-3.79)**	
			
	Hispanic		1.00
	Non-Hispanic		1.41 (0.75-2.64)
			
**Employment Status**			
Work Status			
	Employed	**2.55 (1.75-3.69)**	
	Unemployed	1.03 (0.54-1.94)	
	Not working	1.00	
Hours worked (hours/week)			
	0	1.00	
	< 20	**2.29 (1.18-4.44)**	
	20 to < 37	**2.47 (1.47-4.41)**	
	37+	**2.51 (1.97-3.20)**	
Supervisor			
	Unemployed/not working	1.00	1.00
	Employed and did not supervise employees	**2.31 (1.79-3.00)**	**1.44 (1.10-1.88)**
	Employed and supervised employees	**4.05 (2.26-7.25)**	**1.94 (1.08-3.46)**
Number of jobs			
	No job	1.00	
	One job	**2.48 (1.90-3.23)**	
	More than one job	**3.13 (1.63-5.98)**	
Employment status			
	Full-time	**2.06 (1.68-2.53)**	
	Part-time	1.00	
			
**Social Contextual Factors**			
Neighborhood safety			
	Unsafe	1.00	
	Safe	1.24 (0.91-1.70)	
Health problems make it difficult to exercise			
	Yes	1.00	
	No	1.13 (0.86-1.49)	
TV use (hours/day)			
	None	0.68 (0.31-1.49)	
	> 0 to 2	1.58 (0.98-2.55)	
	> 2 to 4	**1.50 (1.06-2.12)**	
	> 4 to 6	**1.53 (1.06-2.23)**	
	> 6	1.00	
Role conflicts			
	Yes	1.00	
	No	0.88 (0.69-1.11)	
Social ties/networks			
	Few (0,1)	1.00	
	Many (2-4)	1.76 (0.95-3.26)	
Social support			
	Few (0,1)	1.00	
	Many (2-4)	1.34 (0.64-2.80)	
Role responsibilities			
	0	1.00	
	1	1.20 (0.90-1.62)	
	2	**2.47 (1.49-4.09)**	
	3	**2.57 (1.58-4.19)**	
Role responsibilities			
	0-1		1.00
	2-3		**2.18 (1.31-3.61)**
Social cohesion (6-24)		0.99 (0.94-1.04)	

^*^Boldface indicates statistically significant association.

^†^ Variables found to be significant at the P = .15 level in bivariate analyses were retained for use in multivariable modeling. Multivariable-adjusted models are adjusted for age, poverty level, financial status, education, immigrant status, race/ethnicity, supervisory status, and role responsibilities.

#### Bivariate and Multivariable Analyses for Computer Use

Table 3 displays the odds ratios and associated 95% confidence intervals for the polytomous bivariate models. The dichotomous multivariable model is presented in Table 4. Similar to the findings for computer ownership, all sociodemographic factors, with the exception of gender, perceived financial status, and immigration status, were statistically significant predictors of regular computer use in bivariate analyses. As in the previous analyses, education and race/ethnicity were further dichotomized for the multivariable analyses. Multivariable analyses indicated that participants were more likely to be regular computer users if they were above the poverty level (OR = 2.84, 95% CI = 1.90-4.24), had completed high school (OR = 4.43, 95% CI = 3.04-6.46), were non-Hispanic (OR = 1.94, 95% CI = 1.30-2.91), and were employed and supervised others (OR = 2.41, 95% CI = 1.37-4.22).

**Table 3:** Predicting computer use*

		Bivariate Age-Adjusted OR (95% CI)	
		Regular vs Never	Intermittent vs Never
**Sociodemographics**			
Gender				
	Male	1.00	1.00
	Female	1.47 (0.57-1.77)	1.70 (0.88-3.29)
Poverty level			
	Below poverty level	1.00	1.00
	Above poverty level	**3.77 (2.81-5.06)**	0.95 (0.52-1.75)
Financial status				
	Comfortable/enough	1.00	1.00
	Have to cut back/can’t make ends meet	0.97 (0.76-1.23)	**1.40 (1.00-1.96)**
Education			
	≤ 8th grade	1.00	1.00
	Some high school	**3.25 (1.84-5.75)**	**2.30 (1.28-4.15)**
	Completed high school/vocational	**8.61 (4.50-16.46)**	**4.44 (2.23-8.85)**
	At least some college	**38.30 (17.66-83.04)**	**11.19 (5.30-23.63)**
Immigrant			
	No	1.09 (0.59-2.01)	0.91 (0.53-1.55)
	Yes	1.00	1.00
English 1st language				
	No	1.00	1.00
	Yes	**3.07 (2.15-4.38)**	**2.99 (2.05-4.37)**
Race/ethnicity			
	Hispanic	1.00	1.00
	Black	**3.82 (2.75-5.32)**	**3.78 (2.41-5.92)**
	White	**5.64 (3.10-10.26)**	1.64 (0.63-4.28)
	Other	**4.67 (2.61-8.34)**	**2.71 (1.33-5.51)**
			
**Employment Status**			
Work status			
	Employed	**3.60 (2.04-6.37)**	**1.69 (1.00-2.85)**
	Unemployed	**2.07 (1.25-3.44)**	**2.37 (1.29-4.33)**
	Not working	1.00	1.00
Hours worked (hours/week)			
	0	1.00	1.00
	< 20	1.58 (0.56-4.46)	1.36 (0.64-2.92)
	20 to < 37	**2.86 (1.74-4.71)**	1.20 (0.63-2.31)
	37+	**3.60 (2.31-5.60)**	1.51 (0.72-3.18)
Supervisor			
	Unemployed/not working	1.00	1.00
	Employed and did not supervise employees	**2.47 (1.51-4.06)**	1.23 (0.70-2.16)
	Employed and supervised employees	**10.17 (5.80-17.85)**	**3.12 (1.54-6.30)**
Number of jobs			
	No job	1.00	1.00
	One job	**2.85 (1.81-4.48)**	1.33 (0.80-2.20)
	More than one job	**6.07 (2.09-17.61)**	1.89 (0.23-15.58)
Employment status			
	Full-time	**2.95 (2.29-3.80)**	1.35 (0.77-2.38)
	Part-time	1.00	1.00

**Social Contextual Factors**			
Neighborhood safety			
	Unsafe	1.00	1.00
	Safe	**2.19 (1.41-3.40)**	1.60 (0.87-2.93)
TV use (hours/day)			
	0-2	1.30 (0.83-2.05)	0.64 (0.34-1.21)
	> 2 to 6	**1.41 (1.04-1.91)**	0.69 (0.39-1.22)
	> 6	1.00	1.00
Health problems make it difficult to exercise			
	Yes	1.00	1.00
	No	**1.44 (1.17-1.76)**	1.08 (0.75-1.55)
Role conflicts			
	Yes	1.00	1.00
	No	1.01 (0.67-1.51)	1.04 (0.64-1.71)
Social ties/networks			
	Few (0,1)	1.00	1.00
	Many (2-4)	**4.01 (2.42-6.64)**	1.51 (0.74-3.08)
Social support			
	Few (0,1)	1.00	1.00
	Many (2-4)	1.52 (0.67-3.49)	0.91 (0.26-3.15)
Role responsibilities			
	0	1.00	1.00
	1	1.36 (0.76-2.43)	1.07 (0.54-2.10)
	2	1.20 (0.62-2.32)	0.92 (0.50-1.69)
	3	1.78 (0.69-4.68)	1.82 (0.77-4.28)
Social cohesion (1-4)		1.00 (0.85-1.17)	1.12 (0.88-1.42)

^*^Boldface indicates statistically significant association.

**Table 4:** Predicting computer use (regular versus intermittent/never use)*

		Multivariable Age-Adjusted OR (95% CI)^† ^(N = 1210)
**Sociodemographics**		
Poverty level		
	Below poverty level	1.00
	Above poverty level	**2.84 (1.90-4.24)**
Education		
	Did not complete high school	1.00
	Completed high school	**4.43 (3.04-6.46)**
Race/ethnicity		
	Hispanic	1.00
	Non-Hispanic	**1.94 (1.30-2.91)**
		
**Employment Status**		
Supervisor		
	Unemployed/not working	1.00
	Employed and did not supervise employees	1.38 (0.89-2.13)
	Employed and supervised employees	**2.41 (1.37-4.22)**
		
**Social Contextual Factors**		
Neighborhood safety		
	Unsafe	1.00
	Safe	**1.57 (1.08-2.30)**
Social ties/networks		
	Few (0,1)	1.00
	Many (2-4)	**3.09 (1.26-7.59)**

^*^Boldface indicates statistically significant association.

^†^Variables found to be significant at the P = .15 level in bivariate analyses were retained for use in multivariable modeling. Multivariable-adjusted models are adjusted for age, poverty level, financial status, education, immigrant status, race/ethnicity, supervisory status, and role responsibilities.

## Discussion

Computers and the Internet show substantial promise for increasing participation in health promotion activities; thus, we might have more difficulty reducing health disparities if access to technology is not actively promoted [34]. This large study of low-income public housing residents indicated that more than half owned home computers. This level of computer ownership is higher than that found for the general population in recent national surveys (~ 44%) [6] and that found by other studies in similar low-income populations [35].

Most of the attention on reducing the digital divide has been focused on improving access for racial/ethnic minorities. However, access is only one piece of the equation. To fully realize the benefits of computers and the Internet, regular computer use, which builds computer literacy and instills confidence, must be achieved. Our study showed that 42% of participants regularly used a computer, which indicates that there is a large group of low-income racial/ethnic minorities that are potentially experienced computer users. Most participants reported that they used a computer more often at home rather than at work or elsewhere. The location where an individual uses the computer often reflects the quality of their computer and/or computer access [36]. Our finding of substantial home use is encouraging. Most people use computers at home, likely due to a combination of convenience and employment in settings without computer access. Healthy People 2010 emphasizes the importance of home computers and Internet access to increase opportunities for health communication and improve health [37]. Therefore, making computer ownership more available and affordable is important.

This study also points out that there is still a significant group that does not have access to this technology, with 48% of participants reporting that they had never used a computer. The factors that may impact computer use in this population are not clear. Social contextual factors were not as strongly associated with ownership and use as we hypothesized. In addition to employment, we conjecture that cost is likely an issue, as is lack of interest and relevance. Age did appear to be a key factor, in that the majority of older adults (65+ years) did not own (82%) and had never used (86%) a computer. Although older adults are more likely to report greater barriers (eg, vision problems or other disability) to computer use, [38] studies also show that they are just as likely as younger populations to be interested in using computers to look for health information [39,40]. The current computer skills acquired by today’s baby boomers are likely to lessen or eliminate the differential in computer use among the elderly; however, exploring psychosocial and motivational reasons for computer ownership, particularly among elderly who do not currently possess such skills, is important if we are to increase access in this population.

As expected and consistent with the findings of other reports, [36,41,42] we found that sociodemographic factors, employment, and income were positively associated with computer ownership and regular computer use. However, the association between perceived financial hardship and greater ownership would seem counterintuitive considering the above association. We suggest future work to explore whether (1) people now consider computers a necessity and thus find ways to include them in their budget, as noted by one report [43], (2) the association is being confounded by having children under the age of 18 in the household (this was not included in our survey), (3) perceived financial hardship and objective measures of poverty are not measuring the same constructs, or (4) low-income families own computers through the efforts of computer donation programs.

Interestingly, being non-Hispanic was positively associated with regular computer use, but not computer ownership in multivariable models. Although rates of ownership may be similar among racial/ethnic groups, computer use varies. In our study, more Hispanics (61%) than any other racial/ethnic group never used a computer. We also found that greater education was associated with greater computer ownership and regular computer use; in bivariate analyses, there was a positive dose-response relationship between education and ownership and use. Education is a consistently strong predictor of access to and interest in information services, including the Internet and computers [3,6,36,44]. Low levels of education likely explain a large part of the difference in the digital divide between Hispanics and others [45,46], and more strategies are needed to increase ownership and access in this group. Moreover, computers serve as an educational tool that can help increase education for both children and adults [47]. Consequently, the US government and other private nonprofit groups, such as the Bill and Melinda Gates Libraries Initiative, have focused on increasing computer access for low-income groups, such as through public libraries, which are key venues for increasing literacy and an educated workforce [48,49].

Select social contextual factors were also associated with computer ownership and use. For example, feeling safe in one’s neighborhood was associated with a 76% increase in being a regular computer user. This may be particularly salient for those who access computers outside the home, such as a library or neighborhood center. In our study area, there are a number of community computer centers, and this trend of having computers at community centers is growing nationally. We also found that having multiple responsibilities was strongly correlated with computer use. This could be explained by the fact that our low-income study population was largely female (71%) and unmarried (68%) and thus likely to be responsible for childrearing, finances, and taking care of other households (eg, parents). They are also likely to be employed in order to meet these needs. This accounts for our finding that employment increases computer ownership. Moreover, one study found that employed women with caregiving responsibilities were likely to have a “management style” of executing tasks [50]; computers would help them coordinate services and resources to maintain control. We also found an association between social network ties and frequency of computer use. Computer access has been shown to be particularly important in building social support among those dealing with chronic illness. Several studies have shown that online support groups for breast cancer survivors and parents of ill children have positive health and social impacts [51-53]. Stronger social network ties may also provide better access to computers and information about beneficial health programs [54].

### Strengths and Limitations

Our study focused on access to computers among low-income minority groups. We did not specifically ask about Internet access and use. Information regarding Internet use, type of Internet connection, and reasons for computer use would have further contextualized the communication experience of low-income minority adults. However, government reports show that about two thirds of households with computers also have Internet access [41]; rates of Internet access among our participants are therefore likely similar. Also, ownership does not imply use. It is possible that other people in the household (eg, children) actually use the computer. As our study was a cross-sectional design, we were unable to determine whether sociodemographic and social contextual factors causally influence computer ownership, frequency of use, and location. Because this study was conducted in urban, low-income minority public housing communities, its findings are only generalizable to similar settings. Nevertheless, this large study illustrates the level of access to computers among low-income, urban minority adults.

### Conclusion

The racial/ethnic and socioeconomic disparities in access to communication technologies are narrowing, even among very low-income households, making communication technologies for health communication more feasible. However, as the number of technology-based prevention interventions that provide important health information increases, it will be imperative to continue to identify factors that contribute to disparities in access and to connect low-income racial/ethnic minorities to these technologies, particularly computers. In this study, computer ownership among low-income minorities was over 50%, showing noteworthy strides. This suggests that computer-based studies might be reasonable for this population provided that options for nontechnology modalities are also provided. While sociodemographic factors are commonly associated with computer access, a unique finding of our study is that it may be equally as important to consider specific social contextual factors when trying to increase access and use among low-income minorities, such as social network ties, household responsibilities, and neighborhood safety.
